# *Wnt1* Lineage Specific Deletion of *Gpr161* Results in Embryonic Midbrain Malformation and Failure of Craniofacial Skeletal Development

**DOI:** 10.3389/fgene.2021.761418

**Published:** 2021-11-23

**Authors:** Sung-Eun Kim, Karla Robles-Lopez, Xuanye Cao, Kristyn Liu, Pooja J. Chothani, Nikitha Bhavani, Lauren Rahman, Saikat Mukhopadhyay, Bogdan J. Wlodarczyk, Richard H. Finnell

**Affiliations:** ^1^ Department of Pediatrics, Dell Pediatric Research Institute, University of Texas at Austin Dell Medical School, Austin, TX, United States; ^2^ Center for Precision Environmental Health, Department of Molecular and Cellular Biology, Baylor College of Medicine, Houston, TX, United States; ^3^ Department of Cell Biology, University of Texas Southwestern Medical Center, Dallas, TX, United States; ^4^ Departments of Molecular and Human Genetics and Medicine, Baylor College of Medicine, Houston, TX, United States

**Keywords:** Gpr161, midbrain, craniofacial defects, neural crest cells, encephaloceles, sonic hedgehog signaling, Wnt signaling

## Abstract

Sonic hedgehog (Shh) signaling regulates multiple morphogenetic processes during embryonic neurogenesis and craniofacial skeletal development. Gpr161 is a known negative regulator of Shh signaling. Nullizygous Gpr161 mice are embryonic lethal, presenting with structural defects involving the neural tube and the craniofacies. However, the lineage specific role of Gpr161 in later embryonic development has not been thoroughly investigated. We studied the *Wnt1-Cre* lineage specific role of Gpr161 during mouse embryonic development. We observed three major gross morphological phenotypes in *Gpr161* cKO (*Gpr161 f/f; Wnt1-Cre*) fetuses; protrusive tectum defect, encephalocele, and craniofacial skeletal defect. The overall midbrain tissues were expanded and cell proliferation in ventricular zones of midbrain was increased in *Gpr161* cKO fetuses, suggesting that protrusive tectal defects in *Gpr161* cKO are secondary to the increased proliferation of midbrain neural progenitor cells. Shh signaling activity as well as upstream Wnt signaling activity were increased in midbrain tissues of *Gpr161* cKO fetuses. RNA sequencing further suggested that genes in the Shh, Wnt, Fgf and Notch signaling pathways were differentially regulated in the midbrain of *Gpr161* cKO fetuses. Finally, we determined that cranial neural crest derived craniofacial bone formation was significantly inhibited in *Gpr161* cKO fetuses, which partly explains the development of encephalocele. Our results suggest that Gpr161 plays a distinct role in midbrain development and in the formation of the craniofacial skeleton during mouse embryogenesis.

## Introduction

Sonic hedgehog (Shh) signaling is one of the critical mammalian morphogen signaling pathways that regulates dorsoventral neural tube patterning (Cohen et al., 2013), neural stem cell proliferation (Ho and Scott, 2002) and neural crest cell survival (Ahlgren and Bronner-Fraser, 1999) in the developing embryo. Shh is secreted from the notochord and floor plate and plays a critical role for ventral neural tube patterning. Wnts and Bone morphogenetic proteins (BMPs), secreted from the roof plate, modulate the dorsal neural tube patterning. Together, they fine tune the neuronal cell fates during neurulation (Le Dreau and Marti, 2012; Cohen et al., 2013). In addition, Shh signaling is known to have a critical role in embryonic neurogenesis, specifically, neural precursor cell proliferation and differentiation during forebrain and midbrain development (Ho and Scott, 2002; Feijoo et al., 2011; Komada, 2012). Shh signaling also fine-tunes limb patterning (Johnson et al., 1994) and craniofacial development (Xavier et al., 2016) during early fetal life. Therefore, the abnormal regulation of Shh signaling secondary to genetic mutations in the mouse and human results in various congenital malformations, such as neural tube defects (NTDs) (Wu et al., 2013; Lu et al., 2014; Kim et al., 2019; Renard et al., 2019), abnormal brain development (Dhekne et al., 2018; Nagai-Tanima et al., 2020), craniofacial abnormalities (Xavier et al., 2016; De Mori et al., 2017; Wang et al., 2017), and limbs defects (Anderson et al., 2012).

Mammalian neural crest cells (NCCs) arise from the dorsal neural tube which delaminate from their site of origin and subsequently migrate and differentiate into the designated cell types in peripheral organs (Le Douarin and Dupin, 2003; Bronner and Simoes-Costa, 2016). Several kinds of NCCs, such as cranial, vagal, trunk, and sacral NCCs exist, based on their anatomical origins. The cranial NCCs emanate from the diencephalon, mesencephalon, or hindbrain to form the intramembranous craniofacial skeletal elements, including the cranial vaults and jawbones, cranial ganglion, and teeth (Santagati and Rijli, 2003). In particular, the cranial NCCs derived from the diencephalon and mesencephalon forms the craniofacial skeleton in mammals (Kuratani et al., 1997). The spatiotemporal specification of cranial NCCs is tightly regulated by multiple signaling pathways, such as Sonic hedgehog (Shh), Wnt, BMPs, Fibroblast growth factors (Fgfs), and Retinoic acid (RA) (Bronner and Simoes-Costa, 2016). Shh morphogens are secreted from the neuroectoderm of the ventral forebrain, facial ectoderm, and pharyngeal endoderm during early head formation (Nasrallah and Golden, 2001). Shh signaling is also involved in the survival of cranial NCCs (Ahlgren and Bronner-Fraser, 1999). Therefore, mutant mice in Shh signaling, such as the transducer Smoothened (Jeong et al., 2004), Suppressor of Fused (Sufu) (Li et al., 2017) and Fuz (Zhang et al., 2011; Tabler et al., 2016), have been reported to express craniofacial malformations. In humans, genes in the Shh signaling pathway, such as SUFU, are associated with craniofacial and skeletal defects, as is the case with Joubert syndrome (De Mori et al., 2017).

Gpr161 is an orphan G protein-coupled receptor (Matteson et al., 2008) and is a negative regulator of Shh signaling (Mukhopadhyay et al., 2013). It is localized in the primary cilia and activates Protein kinase A (PKA) by increasing cyclic Adenosine monophosphate (cAMP) levels to promote Gli3 processing, thereby inhibiting the Shh target gene expression without the Shh signal. The *Gpr161* hypomorphic mutant mice had both congenital cataracts and spina bifida (Wilson and Wyatt, 1986, 1993; Li et al., 2015). *Gpr161* knockout mice are embryonic lethal by E10.5 and present with NTDs, craniofacial defects, and defective limb buds at E9.5 or E10 (Mukhopadhyay et al., 2013; Kim et al., 2019). The limb buds and facial mesenchyme specific deletion of *Gpr161* in mice results in polysyndactyly and defects of endochondral and intramembranous bone formation in a cilia-dependent manner (Hwang et al., 2018). Neural stem cell-specific deletion of *Gpr161* in mice manifests forebrain phenotypes such as ventriculomegaly, periventricular nodular heterotopia and altered neocortical cytoarchitectonic structure (Shimada et al., 2019), and cerebellar tumors such as Shh-subtype medulloblastoma (Shimada et al., 2018). A nonciliary but cAMP signaling competent *Gpr161* mutant allele is associated with craniofacial abnormalities (Hwang et al., 2021). *GPR161* genetic mutations in humans are also associated with an increased risk for NTDs (Kim et al., 2019) and the pituitary stalk interruption syndrome (Karaca et al., 2015). However, whether Gpr161 plays a role in neural crest cell-derived morphogenesis during embryonic development has not previously been confirmed experimentally.

In this study, we utilized *Wnt1-Cre* transgenic mice to investigate the role of Gpr161 on neural crest lineage specification during murine embryonic development. The *Gpr161* deletion in the *Wnt1-*lineage resulted in the midbrain protrusion and the defects of craniofacial skeletal development. Our results shed new insight into just how Gpr161 regulates *Wnt1-Cre* lineage-specific morphogenesis and skeletogenesis in mice.

## Results

### The Conditional *Gpr161* Deletion in Cranial Neural Crest Lineage Resulted in Midbrain Protrusion and Craniofacial Defects With Encephalocele

**FIGURE 1:**
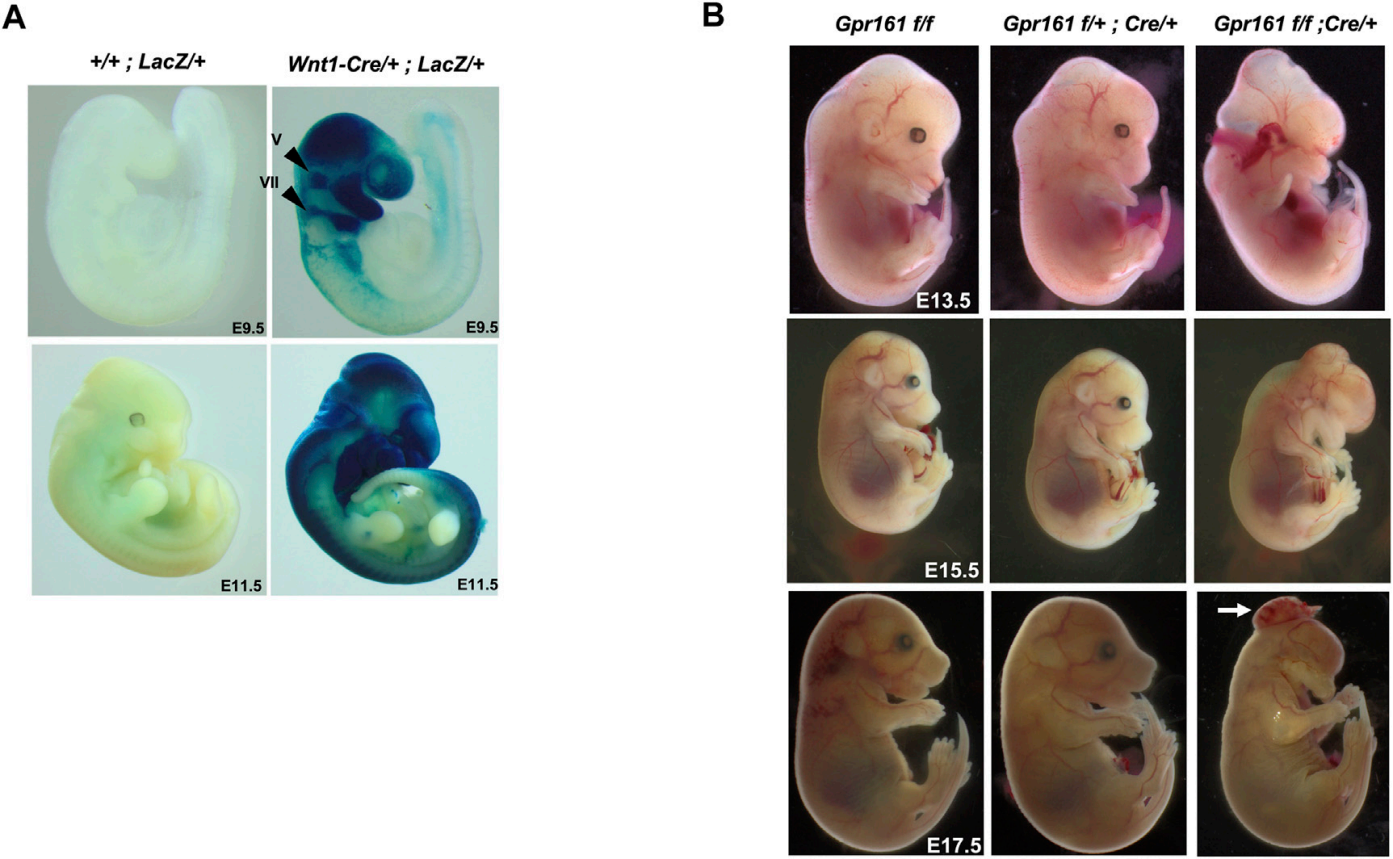
The *Wnt1* lineage specific deletion of *Gpr161* results in protrusive tectum defects and craniofacial defects. **(A)** X-gal staining of *Rosa-LacZ;Wnt1-Cre/+* and *Rosa-LacZ* at E9.5 and E11.5. V: trigeminal ganglia, VII: and facial nerve ganglia **(B)** Gross morphology of *Gpr161* ^*f/f*^, *Gpr161* ^*f/+*^ *;Wnt1-Cre/+*, *Gpr161* ^*f/f*^ *;Wnt1-Cre/+* (*Gpr161*cKO) at E13.5, 15.5 and 17.5. The white arrow indicates encephalocele in E17.5.

The phenotypes of *Gpr161* KO embryos were varied yet included malformations of the pharyngeal arches and microcephaly, along with cranial and spinal NTDs (Mukhopadhyay et al., 2013; Kim et al., 2019). This pattern of altered development suggests that the craniofacial defects could occur in the later embryonic stages. We utilized *Wnt1-Cre* lines to investigate the role of Gpr161 on mouse neural crest-derived craniofacial development in the mouse. We initially characterized the *Cre* expression of *Wnt1-Cre* lines crossed with *Rosa-LacZ* reporter mice [Figure 1A; upper panel)]. *Cre* was expressed in the mesencephalon, the first and second pharyngeal arches, the trigeminal ganglia (V), and facial nerve ganglia (VII) at E9.5. By E11.5, the *Cre* expression was widely expanded into the neural tube, including the mid/hindbrain and most of the orofacial and pharyngeal arch regions (Figure 1A: lower panel). We observed the gross morphology of fetuses with *Wnt1-Cre* lineage-specific *Gpr161* deletion, which resulted from *Gpr161* ^*f/f*^ crossed with *Gpr161* ^*f/+*^ *; Wnt1-Cre/+* (*Gpr161* ^*f/f*^ *; Wnt1-Cre/+*, referred to as *Gpr161* cKO from here on in this manuscript) (Figure 1B). The *Gpr161* cKO fetuses survived until E18.5, although we failed to observe any liveborn pups. The *Gpr161* cKO fetuses expressed midbrain protrusion, ano/microphthalmia, ano/microtia, and severe orofacial defects at E13.5, whereas *Gpr161* ^*f/+*^ *;Wnt1-Cre/+* (*Cre* control) or *Gpr161* ^*f/f*^ (*flox* control) did not show any similar abnormal phenotypes (Figure 1B and Supplementary Table S1). Spinal edema was also apparent by E15.5 in *Gpr161* cKO fetuses. In addition, we could observe widened mandible and maxilla, which are the representative phenotypes of increased Shh signaling in the face (Supplementary Figure S1A). We also observed encephalocele in ∼69% of the *Gpr161* cKO fetuses at E17.5 and E18.5 (Figure 1B and Supplementary Table S2).

**FIGURE 2:**
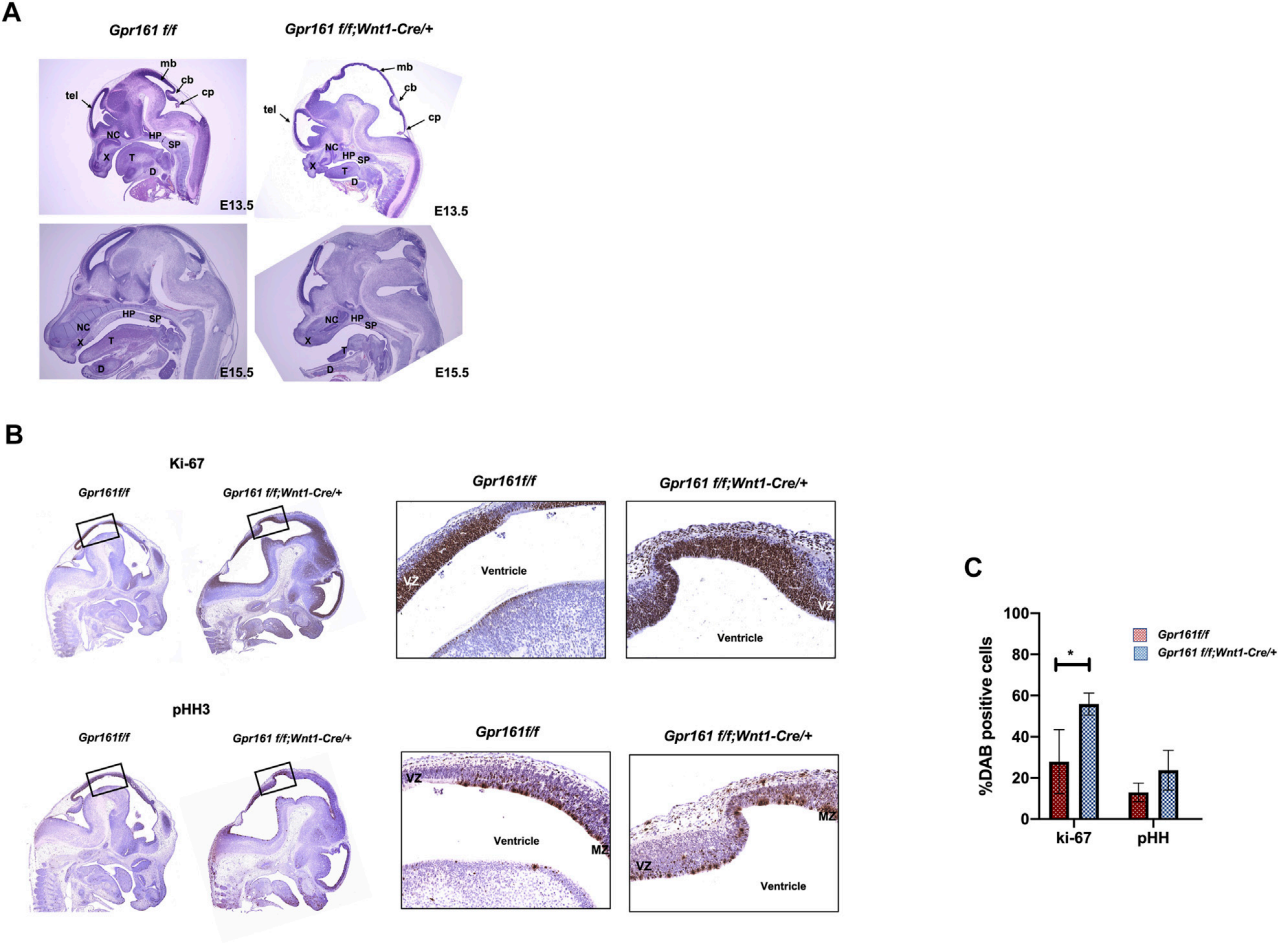
Histological analysis and IHC demonstrating the increased cellular proliferation in the midbrain of *Gpr161* cKO fetuses. **(A)** H&E staining of *Gpr161* ^*f/f*^ and *Gpr161* ^*f/f*^ *;Wnt1-Cre/+* at E13.5 (n = 3) and E15.5 (n = 3); cb, cerebellum; cp, choroid plexus; D, mandible; HP, hard palate; mb, midbrain; NC, nasal cavity; SP, soft palate; T, tongue; tel, telecephalon; X, maxilla **(B)** IHC with Ki-67 and pHH3 antibodies in midbrain sections of *Gpr161* ^*f/f*^ and *Gpr161* ^*f/f*^ *;Wnt1-Cre/+* at E13.5 (n = 3). The black boxes indicate the areas magnified in the right panel. VZ: ventricular zone, MZ: mitotic zone **(C)** The statistical analysis of IHC with Ki-67 and pHH3 shown in **(B)**. *Y* axis indicates the percentage of neurons that were Ki-67, pHH3, and Gli1 positive. The experiments were done triplicate and values were shown as means and standard deviations (SD). The statistical analysis was performed using a 2-way ANOVA, followed by Tukey’s test for multiple comparison (GraphPad Prism 8).

A histological analysis was performed to further confirm the gross phenotypic malformations of the *Gpr161* cKO fetuses (Figure 2A). The tectum in *Gpr161* cKO was extended and mesencephalic vesicle and fourth ventricle at E13.5 were enlarged. The dorsal midbrain was enlarged by E15.5 (Figure 2A: lower right) and the brain herniation along with protruded meninges, which is the representative phenotypes in encephaloceles (Naidich et al., 1992), was detected in the mesencephalic ventricles of *Gpr161* cKO fetuses at E17.5 (Supplementary Figure S1). The maxillary bone was shortened and showed irregular shapes, and nasal septum and mandible were underdeveloped. In addition, hard palatal shelves were not fused along the midline, creating a mild cleft palate in *Gpr161* cKO fetuses at E15.5 and E17.5 (Figure 2A: lower right and Supplementary Figure S1B). These morphological findings were consistent with the phenotypes of the *Gpr161* cKO that were grossly examined in terms of structural malformations of the craniofacies and the mesencephalon.

### Protrusive Tectum Defects Result From Increased Neural Progenitor Cell Proliferation in *Gpr161* cKO Fetuses

As one of the characteristic phenotypes of *Gpr161* cKO fetuses was a midbrain protrusion initially observed at E13.5 and at E15.5, we sought to investigate the underlying cellular defects responsible for this abnormal phenotype. We were interested in clarifying whether these midbrain protrusive phenotypes are primarily due to the ectopic *Wnt1* overexpression or not, as was previously reported (Lewis et al., 2013). We observed that *Cre* control fetuses failed to express any similar midbrain protrusive phenotypes as were seen in the *Gpr161* cKO fetuses (Figure 1B). In addition, *Gpr161* ^*f/f*^ *;Nestin-Cre* fetuses showed similar protrusive tectal defects at E13.5 (Supplementary Figure S2 and Supplementary Table S3) although the phenotypes are less severe than that of *Gpr161* cKO. Both supported that protrusive tectal phenotypes in *Gpr161* cKO resulted from *Gpr161* deletion.

As H&E staining provided evidence of the increased cell proliferation in the midbrain regions in *Gpr161* cKO (Figure 2A), we performed immunohistochemistry (IHC) with Ki67 and pHH3 markers to affirm that the proliferation was regulated in *Gpr161* cKO at E13.5. The Ki67 positive cells in the midbrain regions of *Gpr161* cKO fetuses were significantly increased compared to those of the controls (*Gpr161* ^*f/f*^) (Figures 2B,C), whereas pHH3 positive cells trended towards being increased in the midbrain regions of *Gpr161* cKO fetuses but were not statistically significant. Interestingly, the Ki67 positive cells were widely spread in the ventricular zone (VZ). The pHH3 positive cells tend to be located in the mitotic zone of VZ (Arimura et al., 2019) in WT whereas they were more widely spread out in VZ of the dorsal midbrain in *Gpr161* cKO fetuses (Figure 2B). In addition, we observed the increased Gli1 expression in the dorsal midbrain of *Gpr161* cKO fetuses (Supplementary Figure S3).

### Shh and Wnt Signaling are Involved in the Etiology of Protrusive Tectum Defects in *Gpr161* cKO Fetuses

**FIGURE 3:**
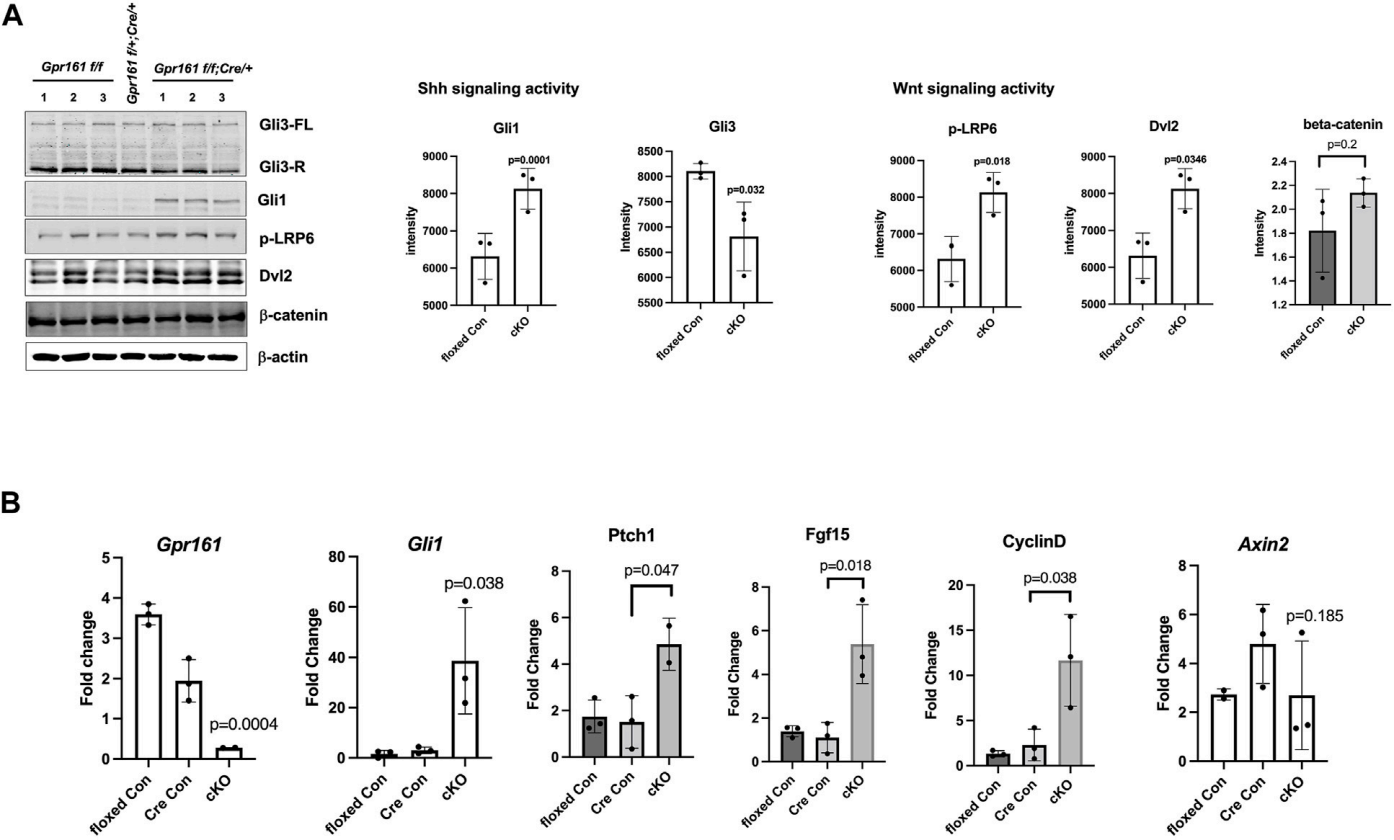
The Shh and Wnt signaling activities in the midbrain of *Gpr161* cKO fetuses at E13.5. The dissected midbrain tissues from *floxed* control (*Gpr161* ^*f/f*^), *Cre* control (*Gpr161* ^*f/+*^ *;Wnt1-Cre/+*) and cKO (*Gpr161* ^*f/f*^ *;Wnt1-Cre/+*) fetuses at E13.5 were used for Western Blotting (WB) and qRT-PCR **(A)** Shh and Wnt signaling activities measured by WB (left). The intensity of each blot was normalized by β-actin. The quantitative analysis (n = 3) (right). Gli3 quantitation was done with repressor forms **(B)** qRT-PCR with *Gpr161*, *Gli1*, *Ptch1*, *Fgf15*, *CyclinD*, *Axin2* (n = 3), which mRNA levels were normalized with *Gapdh*.

Sonic hedgehog signaling as well as Wnt signaling are known to regulate midbrain patterning and proliferation (Brault et al., 2001; Bayly et al., 2007; Blaess et al., 2008). Gpr161 is an established negative regulator of sonic hedgehog signaling in multiple developmental contexts (Mukhopadhyay et al., 2013; Hwang et al., 2018; Kim et al., 2019), and is additionally involved in regulating Wnt signaling as well (Li et al., 2015; Kim et al., 2019). To determine if Shh and Wnt signaling are involved in the increased cell proliferation in the protrusive midbrain of *Gpr161* cKO fetuses, we measured Shh and Wnt signaling activities within dissected midbrain tissues from *floxed*/*Cre* controls and *Gpr161* cKO fetuses (Figure 3). The protein levels of Gli1 and the RNA levels of *Gli1*, *Ptch1*, and *Fgf15*, Shh target genes, were increased (Figures 3A,B). The repressor form of Gli3 was decreased, consistent with increased Gli1 levels in *Gpr161* cKO. These results revealed increased Shh signaling activities in the midbrain tissues of *Gpr161* cKO fetuses. Intriguingly, the protein levels of the Wnt signaling molecules, p-LRP6, Dvl2 (both significantly upregulated) and β-catenin (tends to increase, but not statistically significant), were increased, and one of Wnt target genes, *CyclinD1*, was increased (Figures 3A,B). However, classical target gene, *Axin2*, was not significantly changed in *Gpr161* cKO midbrain tissues (Figure 3B). These results suggested that Gpr161 possibly regulated Wnt signaling in multiple ways.

**FIGURE 4:**
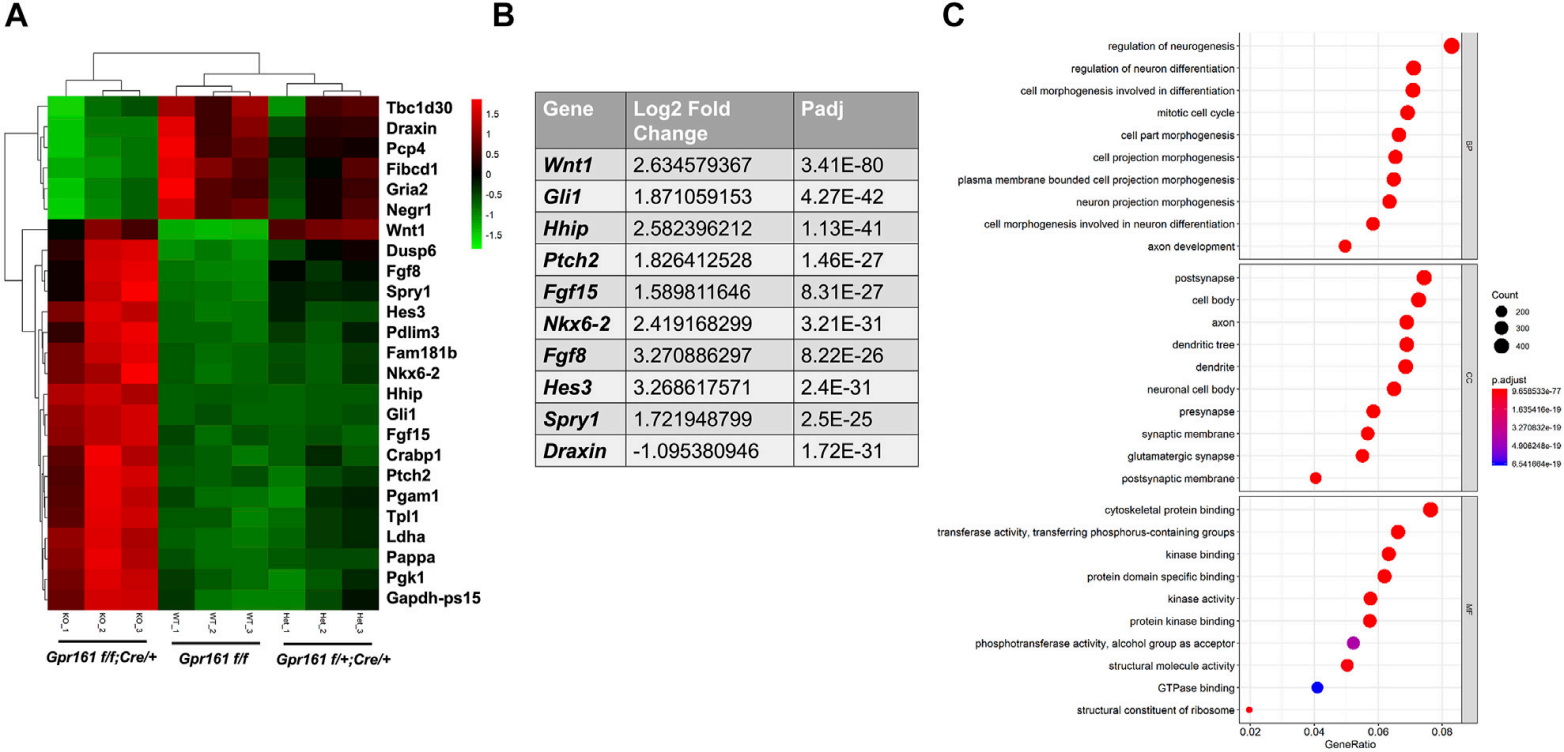
The transcriptomic analysis of the midbrains of *Gpr161* cKO fetuses at E13.5. **(A)** The Heat map from dissected midbrain tissues of *Gpr161* ^*f/f*^ (*floxed* control), *Gpr161* ^*f/+*^ *;Wnt1-Cre/+* (*Cre* control) and *Gpr161* ^*f/f*^ *;Wnt1-Cre/+* (cKO) fetuses at E13.5 (n = 3). Top 25 DEGs were displayed in Heat map (Top 6 downregulated genes in Green in cKO and top 19 upregulated genes in Red in cKO). **(B)** Top ten differentially regulated genes (DEGs) in *Gpr161* cKO **(C)** Gene Ontology (GO) analysis of DEGs in *Gpr161* cKO.

To further unbiasedly identify the molecular basis of protrusive tectum phenotypes in *Gpr161* cKO fetuses, we performed RNA sequencing analysis using midbrain tissues of *Gpr161* ^*f/f*^ (*floxed* control), *Gpr161* ^*f/+*^ *;Cre/+* (*Cre* control), and *Gpr161* cKO (*Gpr161* ^*f/f*^ *;Cre/+*) fetuses at E13.5 (Figure 4). The heatmap showed that the *floxed* control and the *Cre* control had a similar gene expression pattern, except *Wnt1* as the *Cre* control had a higher *Wnt1* expression compared to *floxed* control (Figure 4A). The top 10 differentially expressed genes (DEGs) in the midbrain tissues of *Gpr161* cKO fetuses included: *Gli1*, *Hhip*, *Ptch2*, *Nkx6-2*, *Fgf15* (Shh target genes-upregulated), *Fgf8*, *Spry1* (Fgf signaling related genes-upregulated), *Hes3* (Notch signaling related gene-upregulated), *Wnt1* (agonist in Wnt signaling-upregulated) and *Draxin* (antagonist in Wnt signaling-downregulated) (Figure 4B). These results demonstrated that there was increased Shh, Wnt, as well as Fgf and Notch signaling in the midbrain regions of *Gpr161* cKO fetuses. The Gene Ontology (GO) analysis further demonstrated that DEGs were highly enriched in the processes involved with neurogenesis, neuronal differentiation, neuronal morphogenesis, and mitotic cell cycle (Figure 4C). Taken together, the increased Shh and Wnt signaling are associated with the etiology of protrusive tectum phenotypes found in *Gpr161* cKO.

### The Depletion of *Gpr161* in Cranial Neural Crest Lineages Results in Craniofacial Bone Defects

**FIGURE 5:**
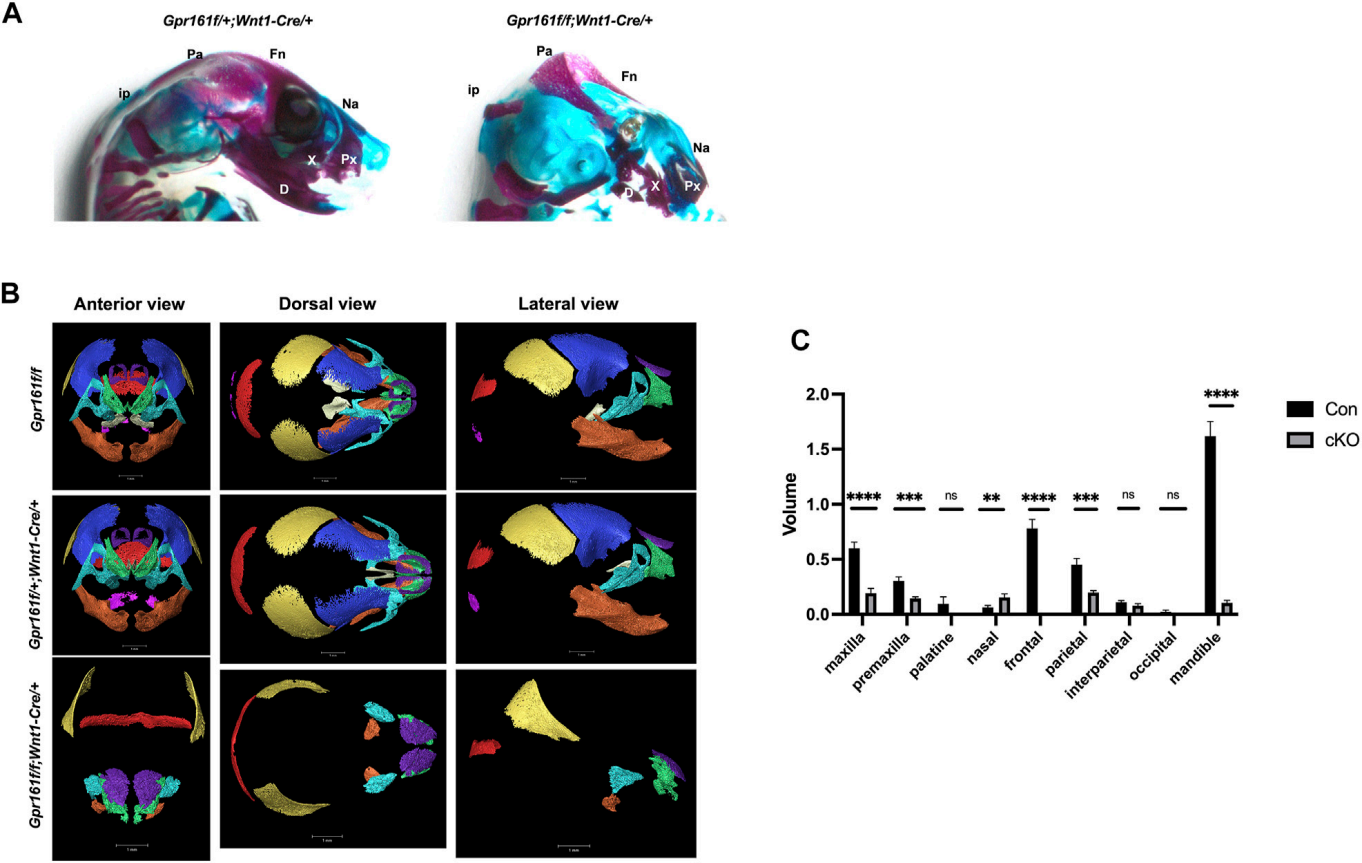
The craniofacial skeletal analysis in *Gpr161* cKO fetuses at E17.5. **(A)** The skeleton staining of heads from *Gpr161* ^*f/+*^ *;Wnt1-Cre/+* and *Gpr161* ^*f/f*^ *;Wnt1-Cre/+* with Alcian blue and Alizarin Red S (n = 3). Fn: frontal bone; Pa: parietal bone; ip: interparietal bone; Na: nasal bone; Px: premaxilla; X: maxilla; D: mandible. **(B)** 3D reconstruction of microCT images of heads from *Gpr161* ^*f/f*^ (n = 1), *Gpr161* ^*f/+*^ *;Wnt1-Cre/+* (n = 2) and *Gpr161* ^*f/f*^ *;Wnt1-Cre/+* (n = 3) at E17.5. frontal bone: blue, parietal bone: yellow, interparietal bone: red, occipital bone: pink, nasal bone: purple, palatine bone: beige, maxilla: turquoise, premaxilla: green, mandible: orange **(C)** Quantitative volume measurement of identified craniofacial bones. Con combines the volume measurement from *Gpr161* ^*f/f*^ and *Gpr161* ^*f/+*^ *;Wnt1-Cre/+*.

The gross morphology (Figure 1B) and histological analysis (Figure 2A) suggested abnormal facial and cranial structures in *Gpr161* cKO fetuses. We observed up to 69% of the *Gpr161* cKO fetuses had encephalocele (Supplementary Table S2). The observed encephalocele could also be secondary to skull defects. To investigate craniofacial bone development in *Gpr161* cKO fetuses, we performed skeletal staining with Alcian Blue (unmineralized cartilages) and Alizarin Red S (mineralized cartilages and bones). We observed a significant loss of mineralized skull and facial bones in *Gpr161* cKO fetuses at E17.5 (Figure 5A). Specifically, the frontal, maxillary, and mandibular bones, which are derived from neural crest cell lineages, were significantly underdeveloped while the frontal bones failed to even form. The formation of parietal bones, which are derived from paraxial mesodermal cell lineages, was also severely reduced.

To further validate the skeletal staining results, we performed a bone segmentation study using 3D micro-CT imaging and further measured the volume of each bone based on micro-CT data within the craniofacial regions of *floxed* control, *Cre* control and *Gpr161* cKO fetuses at E17.5 (Figures 5B,C). We failed to observe any overall head size differences between controls and *Gpr161* cKO fetuses. Consistent with the findings of the skeletal staining studies, the neural crest lineage derived bones in the cranial vault and facial bones, including the maxilla, premaxilla, mandible, and frontal, were significantly underdeveloped or completely absent (Figure 5B) and their volumes were significantly reduced in *Gpr161* cKO fetuses (Figure 5C). However, palatine bone formation was not changed, and the volume of nasal bones was increased in *Gpr161* cKO fetuses. Additionally, segments and volume of the parietal bones were significantly reduced as shown in the skeletal staining of the fetuses, whereas the formation of other bones derived from paraxial mesoderm, such as the interparietal and occipital bones, were not affected in *Gpr161* cKO fetuses. These results strongly suggest that Gpr161 has a significant role in the formation of the neural crest derived cranial vault and facial bones.

## Discussion

**FIGURE 6:**
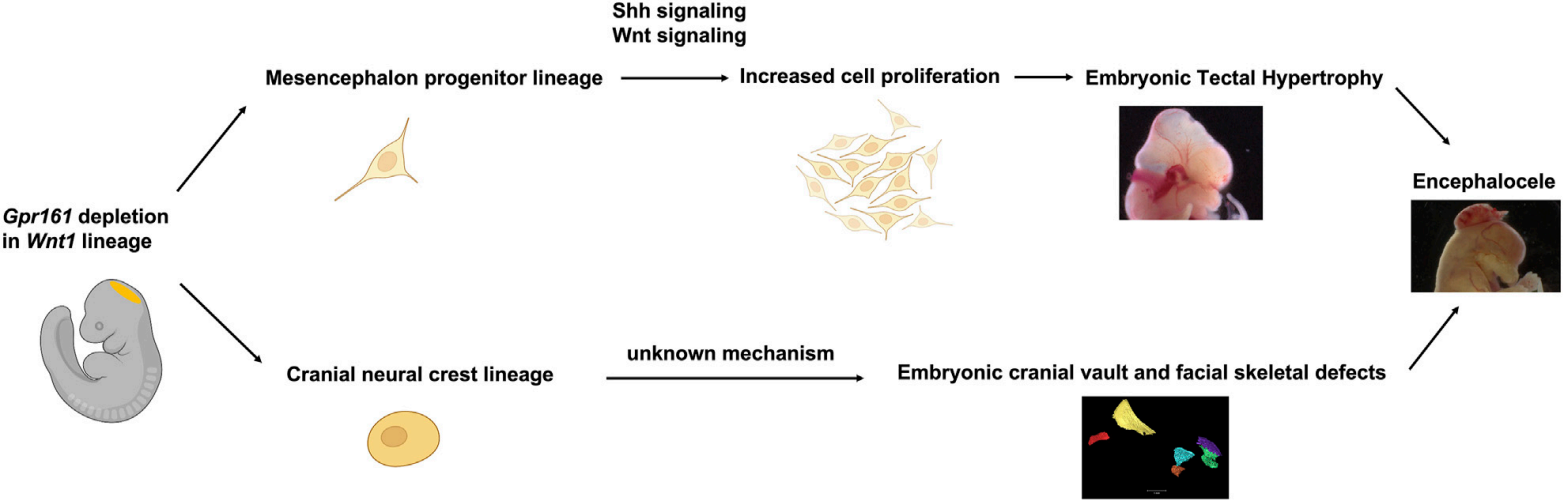
Schematic summary of *Gpr161* depletion in *Wnt1*-lineages in mouse. Midbrain dorsal neuroectoderm derived mesencephalon progenitor with *Gpr161* depletion resulted in the increased cell proliferation via up-regulated Shh and Wnt signaling, thereby contributing to embryonic tectal protrusive phenotypes from E13.5. The *Gpr161* depleted cranial neural crest cells derived from the mesencephalon caused severe cranial vault and facial bone defects. Both embryonic tectal protrusion and craniofacial bone defects could contribute to encephaloceles. This schematic figure was partly created with BioRender.com.

The *Wnt1-Cre* lineage-specific deletion of *Gpr161* in mice resulted in two significant phenotypes; one involves protrusive tectal defects, while the other are craniofacial skeletal defects, both of which may underlie the development of encephalocele in some fetuses. The protrusive tectal defects in *Gpr161* cKO fetuses are partly due to the increased midbrain neural progenitor cell proliferation. The increased proliferation in the midbrain is associated with the elevated Shh signaling due to *Gpr161* deletion and the increased Wnt signaling, as was showed in Western Blot and qRT-PCR assays (Figure 3), as well as RNA seq experiments (Figure 4). On the contrary, the *Gpr161* depletion in neural crest cells caused severe defects in intramembranous bone formation, specifically involving the cranial vault and facial bones (Summarized in Figure 6).

### The Role of Gpr161 on the Embryonic Midbrain Development

As previously reported (Lewis et al., 2013) and shown in Figure 1A, *Wnt1-Cre* driver-mediated recombination initially occurs in the midbrain dorsal neuroectoderm and the neural crest cells derived from mesencephalon forms craniofacial cartilages and bones (Santagati and Rijli, 2003). The brain hypertrophy, specifically protrusive tectal phenotypes in *Gpr161* cKO fetuses with *Wnt1-Cre*, appears as if it is a phenocopy of *Ptch1* cKO with *Nestin-Cre* (Martinez et al., 2013). We also observed identical phenotypes in *Gpr161* cKO with *Nestin-Cre* fetuses (Supplementary Figure S2), indicating the critical role that Gpr161 serves during midbrain morphogenesis. The dorsal midbrain proliferation was increased in both *Gpr161* cKO with *Wnt1-Cre* and *Ptch1* cKO with *Nestin-Cre*, suggesting that Shh signaling is required for the tectal progenitor cell proliferation. Indeed, Shh target gene expression was increased (Figures 3, 4B), and Gli3 processing (Figure 3A) was inhibited in midbrain tissues of *Gpr161* cKO with *Wnt1-Cre*, indicating the increased Shh signaling. In addition, five out of the top ten DEGs in the RNA seq data set were Shh target genes, including *Gli1*, *Hhip*, *Ptch2*, *Fgf15*, and *Nkx6.2* (Figure 4B), which were upregulated in *Gpr161* cKO fetuses, providing further supporting evidence of an increased Shh signaling in the *Gpr161* cKOs. Together, these results explain that increased Shh signaling involved in the increased mesencephalon proliferation, thereby causing the protrusive tectum in the affected fetuses. The forebrain ventricular surface was also reported to be expanded during embryogenesis from radial glial over-proliferation upon *Nestin-Cre* mediated deletion of *Gpr161* (Shimada et al., 2019). Cortical phenotypes that were observed included polymicrogyria in the medial cingulate cortex, increased proliferation of intermediate progenitors and basal radial glia, and altered neocortical cytoarchitectonic structure with increased upper layer and decreased deep layer neurons. Overall results support the role of Gpr161 in the cell proliferation during fore/mid brain morphogenesis. In addition, the protrusive mesencephalon in *RhoA* cKO with *Wnt1-Cre* fetuses results from the hyperproliferation of midbrain progenitor cells via increased Shh signaling, providing yet more evidence supporting the role of Shh signaling in dorsal midbrain progenitor cell proliferation (Katayama et al., 2011). As *Ptch1* cKO with *Nestin-Cre* showed similar midbrain protrusive phentoypes as did *Gpr161* cKO, it will be an interesting future study to identify the relation between *Gpr161* and other Shh signaling molecules (e.g. *Ptch1* or *Smo*) during embryonic midbrain morphogenesis.

Wnt signaling is well known to be associated with mesencephalon development (Brault et al., 2001; Panhuysen et al., 2004; Chilov et al., 2010), and our results further demonstrated that the activated Wnt signaling is also involved in the mesencephalic cell proliferation. The activities of upstream signaling molecules in Wnt signaling, phosphorylated LRP6 and Dvl2, were increased and one of target genes, *CyclinD1*, was increased (Figure 3). At the same time, the expression of Draxin, an inhibitor of canonical Wnt signaling (Miyake et al., 2009), was decreased in *Gpr161* cKO fetuses (Figure 4B), providing evidence that the increased Wnt signaling secondary to *Gpr161* depletion contributes to the observed mesencephalon cell proliferation. However, we cannot rule out the possibility that Wnt and Shh signaling regulate cell proliferation in the midbrain tissues of *Gpr161* cKO in parallel. Additionally, the level of downstream Wnt signaling molecules, such as β-catenin and *Axin2*, was unchanged (Figure 3), maintaining the complexity of the interactions between Shh and Wnt signaling. Nonetheless, the interplay between Shh and Wnt signaling in mesencephalon progenitor cell proliferation and differentiation in *Gpr161* cKO fetuses remains an inadequately resolved question (Tang et al., 2010). Clearly, the molecular interactions between Gpr161, Fgf, Notch, and Wnt signaling requires future investigation.

### The Intramembranous Skeletal Defects With Encephalocele in *Gpr161* cKO Fetuses

*Gpr161* depletion with *Wnt1-Cre* resulted in severe craniofacial skeletal defects (Figure 5). It is notable that the portions of the craniofacial skeleton derived from neural crest cells were completely absent, while those portions derived from mesenchyme were only partially reduced. The partial reduction of parietal bones, which derived from mesodermal lineages, in *Gpr161* cKO could be explained by two possibilities. Jiang et al. (2002) suggests the possibility that the neural crest derived meninges are required for the mesodermal derived bone ossification. The other possibility is the possible Cre expression in non-neural crest cells due to leakage or due to insertion side effect in *Wnt1-Cre* line (Doro et al., 2019).

The *Gpr161* depletion in mesenchymal lineages was reported to include phenotypes with the posterior cranial vault defects caused by the lack of intramembranous ossification (Hwang et al., 2018), supporting the role of *Gpr161* during cranial vault skeletogenesis. One possible explanation regarding craniofacial skeletal defects in *Gpr161* cKOs is that the increased neural crest cell populations due to increased Shh signaling resulting from the *Gpr161* depletion might cause craniofacial skeletal defects, as was previously observed in *Fuz* knockout mice (Tabler et al., 2016). Another possible explanation is the involvement of Wnt/β-catenin signaling as it plays a critical role in the intramembranous bone formation (Day and Yang, 2008). This possibility is supported by the previous report that *β-catenin* cKO embryos with *Wnt1-Cre* was phenocopied in *Gpr161* cKO with *Wnt1-Cre* in terms of their complete lack of neural crest cell derived cranial vault and facial bones (Brault et al., 2001). However, the underlying cellular and molecular processes of craniofacial skeletal defects in *Gpr161* cKO need to be further explored. It will be an additional future study to investigate the relation between *Gpr161* and other Shh signaling molecules, such as *Sufu* or *Smo*, during embryonic craniofacial skeletogenesis.

### Implications of the Role of Gpr161 in Encephaloceles

The pathogenesis and the etiology of the structural malformations known as encephaloceles have not been comprehensively studied even though it is often classified as a type of neural tube defects. A recent publication (Rolo et al., 2019) showed that encephaloceles result from the defective surface ectoderm in a post-neurulation manner along with severe calvarial bone defects at a later embryonic stage in the surface ectoderm specific deletion of *Rac1* mouse model. As *Gpr161* cKO did not show any significant phenotypic malformations at E9.5 or 10.5, the encephaloceles in *Gpr161* cKO clearly occur in post-neurulation stage embryos. In addition, encephaloceles are often observed to be associated with other human birth defects, including other NTDs, cleft palate, craniosynostosis (Naidich et al., 1992; Caplan et al., 2002; Ganapathy et al., 2014), which are similar phenotypes to those observed in mouse models with *Gpr161* cKO or *Rac1* cKO (Rolo et al., 2019). Therefore, our study provides a potential mouse model of encephaloceles, which enables us to further study the molecular pathogenicity of this much-understudied congenital malformation.

### Conclusion and Future Directions

In this study, we attempted to unravel the role of Gpr161 in embryonic midbrain development and craniofacial bone formation in mouse models with *Wnt1* lineage specific deletion of *Gpr161*, demonstrating the distinct role of Gpr161 in mesencephalon proliferation and neural crest derived craniofacial skeleton morphogenesis. Our data suggests that *Gpr161* cKO can serve as a mouse model for enhancing our understanding of the basic developmental biology of encephaloceles. The results from this study also suggest a possible genetic association of *GPR161* with such craniofacial defects as cleft palate, as well as encephaloceles in humans. Based on this study, we will further delineate the role of Gpr161 in neural crest cell differentiation and will also study the genetic association of *GPR161* and associated Sonic Hedgehog genes and craniofacial birth defects in human patient samples.

## Materials and Methods

### Mouse Strains

All mice were housed and handled according to the guidelines approved by the Institutional Animal Care and Use Committee (IACUC) of The University of Texas at Austin. *Gpr161* conditional knock out mice (*Gpr161 flox*) were generated and graciously provided by Dr. Saikat Mukhopadhyay (UT Southwestern, Dallas) and the detailed information was previously reported (Hwang et al., 2018). The transgenic mice, *Wnt1-Cre* (#009107), *Nestin-Cre* (#003771), and *Rosa26-lsl-LacZ* (#003474), were purchased from Jackson laboratory. The genotypes of the mice and embryos/fetuses were determined by PCR-based genotyping.

### Whole Mount β-Galactosidase Staining

Embryos were collected at E9.5 and E11.5 from timed mated breeding pairs between *Wnt1-Cre* and *Rosa26-LacZ*. The harvested embryos were fixed, and β-gal staining was performed according to the manufacturer’s instruction (Millipore Sigma). The images were captured by Leica stereomicroscope with a Nikon digital camera.

### Immunohistochemistry

Fetuses were harvested at either E13.5 or E15.5 from timed mating between *Gpr161* ^*f/f*^ and *Gpr161* ^*f/+*^ *;Wnt1-Cre/+*. Collected fetuses were fixed, paraffin embedded, and sectioned with 4 um-thickness. The paraffin sections were deparaffinized, dehydrated, antigen-retrieved, blocked (blocking solution: Thermo Fisher scientific), and incubated with primary antibodies (Ki67, pHH3, and Gli1) diluted with Lab Vision™ Antibody Diluent Quanto (Thermo Fischer scientific) overnight at 4°C. After washing, sectioned were incubated with Horseradish peroxidase (HRP) polymer conjugate (UnltraVision™ LP detection system, Thermo Fisher scientific) and DAB (Boster Bio). The sections were counterstained with hematoxylin (Thermo Fisher scientific). Images were captured with All-In-One Fluorescent (Keyence) microscope using a ×2 and ×20 objective. To assess the positive cells for each proliferation marker Ki-67 and pHH3 in the dorsal midbrain regions of each embryo, the images were digitized and analyzed with Fiji (NIH), with image analysis. For quantification, a total of 4–6 fields per dorsal midbrain regions per each embryo were captured at ×20 magnification. Each field was divided into 100 equal squares and subjected to color deconvolution. For each of the images, the Shanbhag threshold (Shanbhag, 1994) was applied to DAB-only images. The stained fraction was measured in the threshold fields (color deconvolution also gives 4′6′-diamidino-2-phenylindole (DAPI), and helps identify each threshold cell), then percentage of DAB positive cells from each image was calculated and assessed. The mean of 10 values obtained across from the six fields was calculated for each of the markers and each of the embryos, which then were subjected for the statistical analysis.

### Midbrain Dissection and Western Blot

The midbrain tissues were dissected from E13.5 mouse fetuses as described (Weinert et al., 2015). The dissected midbrain tissues were lysed with Radioimmunoprecipitation assay (RIPA) lysis buffer. The denatured protein samples were immunoblotted using anti-Gli3 (Santa Cruz Biotechnology), Gli1, p-LRP6, Dvl2, β-actin (Cell signaling), β-catenin (BD bioscience) and then with 1RDye^®^ 800CW goat anti-rabbit IgG and 1RDye^®^ 680CW goat anti-mouse IgG secondary antibodies (LI-COR). The images were captured by Odyssey^®^ (LI-COR).

### RNA Extraction and Quantitative RT-PCR

The dissected midbrain tissues from E13.5 mouse fetuses were lysed with Trizol and total RNA was purified with Direct-zol RNA kit (Zymo research). For quantitative RT-PCR, 500 ng-1 µg of RNA was used to synthesize cDNA using iScript reverse transcription Supermix kit (Bio-Rad). The quantitative RT-PCR was performed using SsoAdvanced™ Universal SYB R Green Supermix (Bio-Rad) according to the manufacturer’s instruction. The primers for qRT-PCR are as below.

**Table:** 

	Forward (5′→3′)	Reverse (5′→3′)
*Gpr161*	TCG​GTG​GAG​TTT​GAT​GAG​TTC​A	CCG​TAG​CAC​ACT​AGC​ATG​ATG​A
*Gli1*	CCA​AGC​CAA​CTT​TAT​GTC​AGG​G	AGC​CCG​CTT​CTT​TGT​TAA​TTT​GA
*Ptch1*	TGG​CTC​TTG​GAG​GGC​AGA​AAT​TAC	CCT​GGG​TGG​TCT​CTC​TAC​TTT​GGT
*Fgf15*	GAG​GAC​CAA​AAC​GAA​CGA​AAT​T	ACG​TCC​TTG​ATG​GCA​ATC​G
*CyclinD1*	TCCCAGACGTTCAGAACC	AGG​GCA​TCT​GTA​AAT​ACA​CT
*Axin2*	AAG​TGT​CTC​TAC​CTC​ATT​TTC​CG	TCC​AGT​TTC​AGT​TTC​TCC​AGC
*Gapdh*	GAC​CTG​CCG​TCT​AGA​AAA​AC	CTG​TAG​CCA​AAT​TCG​TTG​TC

### RNA Sequencing

The tissues were harvested as described in Midbrain dissection and total RNAs were extracted with Direct-zol RNA kit (Zymo Research). The quantity and integrity of RNAs were analyzed by Nanodrop (Thermo Fisher) and Bioanalyzer (Agilent Technologies). The library was prepared with NEBNext Ultra RNA with Poly-A selection and (NEB) was sequenced on an Illumina Hi-Seq 4000 (Admera Health LLC). The differential gene expression was determined with fold change >1.5 and *p* < 0.05 genes with <1 count per million (cpm). Any gene with a *p*-value greater than FDR, after Benjamini-Hochberg correction for multi-testing, was deemed significantly differentially expressed under the test condition as compared to the control. The dataset was analyzed by the Gene Ontology (GO) enrichment analysis.

### Bone-Cartilage Skeletal Staining

Skeletal staining was performed using a modified Alcian Blue/Alizarin Red staining procedure (Kessel et al., 1990). Briefly, the E17.5 fetuses were eviscerated and fixed with 95% ethanol and then acetone. Fixed fetuses were incubated with staining solution (0.005% Alizarin red S 0.015% Alcian Blue GS in 5% acetic acid, 5% H_2_O and 90% ethanol) for 3 days at 37°C. After washing, samples were kept in 1% KOH for 48 h. For long term storage, specimens were transferred into 20, 50 and 80% glycerol solutions and were ultimately maintained in 100% glycerol. The images were captured by a Leica stereomicroscope with a Nikon digital camera.

### Micro-CT Scan and Image Processing

The E17.5 fetuses were fixed with 10% formalin followed by 70% ethanol. Specimens were scanned at the University of Texas High-Resolution X-ray CT Facility using the flat panel detector on a Zeiss Xradia 620 Versa. The X-ray source was set to 70 kV and 8.5 W with no filter. A total of 2001 0.1s projections were acquired over ±180 degrees of rotation with no frame averaging. A source-object distance of 18.0 mm and a detector-object distance of 251.7 mm resulted in 9.98-micron resolution. The resulting data were segmented in Avizo software v.2020.2.

### Statistical Analysis

The experiments were done in triplicate Unless specifically stated otherwise and the data was analyzed by the Standard Deviation (SD) with student t-test for comparing groups. For IHC data, the statistical analysis was performed with 2-way ANOVA, followed by Tukey’s test for multiple comparison (GraphPad Prism9).

## Data Availability

The datasets presented in this study can be found in online repositories. The names of the repository/repositories and accession number(s) can be found below: Gene Expression Omnibus GSE185336.

## References

[B1] Ahlgren S. C. Bronner-Fraser M. (1999). Inhibition of Sonic Hedgehog Signaling *In Vivo* Results in Craniofacial Neural Crest Cell Death. Curr. Biol. 9 (22), 1304–1314. 10.1016/s0960-9822(00)80052-4 10574760

[B2] Anderson E. Peluso S. Lettice L. A. Hill R. E. (2012). Human Limb Abnormalities Caused by Disruption of Hedgehog Signaling. Trends Genet. 28 (8), 364–373. 10.1016/j.tig.2012.03.012 22534646

[B3] Arimura N. Dewa K.-i. Okada M. Yanagawa Y. Taya S.-i. Hoshino M. (2019). Comprehensive and Cell-Type-Based Characterization of the Dorsal Midbrain during Development. Genes Cells 24 (1), 41–59. 10.1111/gtc.12656 30422377

[B4] Bayly R. D. Ngo M. Aglyamova G. V. Agarwala S. (2007). Regulation of Ventral Midbrain Patterning by Hedgehog Signaling. Development 134 (11), 2115–2124. 10.1242/dev.02850 17507412

[B5] Blaess S. Stephen D. Joyner A. L. (2008). Gli3 Coordinates Three-Dimensional Patterning and Growth of the Tectum and Cerebellum by Integrating Shh and Fgf8 Signaling. Development 135 (12), 2093–2103. 10.1242/dev.015990 18480159 PMC2673693

[B6] Brault V. Moore R. Kutsch S. Ishibashi M. Rowitch D. H. McMahon A. P. (2001). Inactivation of the (β)-Catenin Gene by Wnt1-Cre-Mediated Deletion Results in Dramatic Brain Malformation and Failure of Craniofacial Development. Development 128 (8), 1253–1264. 10.1242/dev.128.8.1253 11262227

[B7] Bronner M. E. Simões-Costa M. (2016). The Neural Crest Migrating into the Twenty-First Century. Curr. Top. Dev. Biol. 116, 115–134. 10.1016/bs.ctdb.2015.12.003 26970616 PMC5100668

[B8] Caplan J. Angel M. Parent A. (2002). Encephalocele as a Late Complication of Cranial Vault Reconstruction in a Patient with Crouzon's Syndrome. J. Craniofac. Surg. 13 (4), 501–504. 10.1097/00001665-200207000-00004 12140411

[B9] Chilov D. Sinjushina N. Saarimäki-Vire J. Taketo M. M. Partanen J. (2010). Beta-Catenin Regulates Intercellular Signalling Networks and Cell-type Specific Transcription in the Developing Mouse Midbrain-Rhombomere 1 Region. PLoS One 5 (6), e10881. 10.1371/journal.pone.0010881 20532162 PMC2880587

[B10] Cohen M. Briscoe J. Blassberg R. (2013). Morphogen Interpretation: the Transcriptional Logic of Neural Tube Patterning. Curr. Opin. Genet. Dev. 23 (4), 423–428. 10.1016/j.gde.2013.04.003 23725799

[B11] Day T. F. Yang Y. (2008). Wnt and Hedgehog Signaling Pathways in Bone Development. J. Bone Jt. Surg Am. 90 (Suppl. 1), 19–24. 10.2106/JBJS.G.01174 18292352

[B12] De Mori R. Romani M. D’Arrigo S. Zaki M. S. Lorefice E. Tardivo S. (2017). Hypomorphic Recessive Variants in SUFU Impair the Sonic Hedgehog Pathway and Cause Joubert Syndrome with Cranio-Facial and Skeletal Defects. Am. J. Hum. Genet. 101 (4), 552–563. 10.1016/j.ajhg.2017.08.017 28965847 PMC5630196

[B13] Dhekne H. S. Yanatori I. Gomez R. C. Tonelli F. Diez F. Schüle B. (2018). A Pathway for Parkinson's Disease LRRK2 Kinase to Block Primary Cilia and Sonic Hedgehog Signaling in the Brain. Elife 7, e40202. 10.7554/eLife.40202 30398148 PMC6219843

[B14] Doro D. Liu A. Grigoriadis A. E. Liu K. J. (2019). The Osteogenic Potential of the Neural Crest Lineage May Contribute to Craniosynostosis. Mol. Syndromol. 10 (1-2), 48–57. 10.1159/000493106 30976279 PMC6422151

[B15] Feijóo C. G. Oñate M. G. Milla L. A. Palma V. A. (2011). Sonic Hedgehog (Shh)-Gli Signaling Controls Neural Progenitor Cell Division in the Developing Tectum in Zebrafish. Eur. J. Neurosci. 33 (4), 589–598. 10.1111/j.1460-9568.2010.07560.x 21219478

[B16] Ganapathy A. T S. Swer M. H. Rao S. (2014). Occipital Meningoencephalocele with Cleft Lip, Cleft Palate and Limb Abnormalities- A Case Report. J. Clin. Diagn Res. 8 (12), AD03–05. 10.7860/JCDR/2014/10842.5326 PMC4316239 25653933

[B17] Ho K. S. Scott M. P. (2002). Sonic Hedgehog in the Nervous System: Functions, Modifications and Mechanisms. Curr. Opin. Neurobiol. 12 (1), 57–63. 10.1016/s0959-4388(02)00290-8 11861165

[B18] Hwang S.-h. White K. A. Somatilaka B. N. Shelton J. M. Richardson J. A. Mukhopadhyay S. (2018). The G-Protein-Coupled Receptor Gpr161 Regulates Forelimb Formation, Limb Patterning and Skeletal Morphogenesis in a Primary Cilium-Dependent Manner. Development 145 (1), dev154054. 10.1242/dev.154054 29222391 PMC5825871

[B19] Hwang S.-H. Somatilaka B. N. White K. Mukhopadhyay S. (2021). Ciliary and Extraciliary Gpr161 Pools Repress Hedgehog Signaling in a Tissue-specific Manner. Elife 10, e67121. 10.7554/eLife.67121 34346313 PMC8378848

[B20] Jeong J. Mao J. Tenzen T. Kottmann A. H. McMahon A. P. (2004). Hedgehog Signaling in the Neural Crest Cells Regulates the Patterning and Growth of Facial Primordia. Genes Dev. 18 (8), 937–951. 10.1101/gad.1190304 15107405 PMC395852

[B21] Jiang X. Iseki S. Maxson R. E. Sucov H. M. Morriss-Kay G. M. (2002). Tissue Origins and Interactions in the Mammalian Skull Vault. Dev. Biol. 241 (1), 106–116. 10.1006/dbio.2001.0487 11784098

[B22] Johnson R. L. Riddle R. D. Laufer E. Tabin C. (1994). Sonic Hedgehog: a Key Mediator of Anterior-Posterior Patterning of the Limb and Dorso-Ventral Patterning of Axial Embryonic Structures. Biochem. Soc. Trans. 22 (3), 569–574. 10.1042/bst0220569 7821639

[B23] Karaca E. Buyukkaya R. Pehlivan D. Charng W.-L. Yaykasli K. O. Bayram Y. (2015). Whole-exome Sequencing Identifies Homozygous GPR161 Mutation in a Family with Pituitary Stalk Interruption Syndrome. J. Clin. Endocrinol. Metab. 100 (1), E140–E147. 10.1210/jc.2014-1984 25322266 PMC4283017

[B24] Katayama K.-i. Melendez J. Baumann J. M. Leslie J. R. Chauhan B. K. Nemkul N. (2011). Loss of RhoA in Neural Progenitor Cells Causes the Disruption of Adherens Junctions and Hyperproliferation. Proc. Natl. Acad. Sci. 108 (18), 7607–7612. 10.1073/pnas.1101347108 21502507 PMC3088619

[B25] Kessel M. Balling R. Gruss P. (1990). Variations of Cervical Vertebrate after Expression of a Hox-1.1 Transgene in Mice. Cell 61 (2), 301–308. 10.1016/0092-8674(90)90810-2 1970515

[B26] Kim S.-E. Lei Y. Hwang S.-H. Wlodarczyk B. J. Mukhopadhyay S. Shaw G. M. (2019). Dominant Negative GPR161 Rare Variants Are Risk Factors of Human Spina Bifida. Hum. Mol. Genet. 28 (2), 200–208. 10.1093/hmg/ddy339 30256984 PMC6321953

[B27] Komada M. (2012). Sonic Hedgehog Signaling Coordinates the Proliferation and Differentiation of Neural Stem/progenitor Cells by Regulating Cell Cycle Kinetics during Development of the Neocortex. Congenit. Anom. (Kyoto) 52 (2), 72–77. 10.1111/j.1741-4520.2012.00368.x 22639991

[B28] Kuratani S. Matsuo I. Aizawa S. (1997). Developmental Patterning and Evolution of the Mammalian Viscerocranium: Genetic Insights into Comparative Morphology. Dev. Dyn. 209 (2), 139–155. 10.1002/(sici)1097-0177(199706)209:2<139:aid-aja1>3.0.co;2-j 9186050

[B29] Le Douarin N. M. Dupin E. (2003). Multipotentiality of the Neural Crest. Curr. Opin. Genet. Dev. 13 (5), 529–536. 10.1016/j.gde.2003.08.002 14550420

[B30] Le Dréau G. Martí E. (2012). Dorsal-ventral Patterning of the Neural Tube: a Tale of Three Signals. Devel Neurobio. 72 (12), 1471–1481. 10.1002/dneu.22015 22821665

[B31] Lewis A. E. Vasudevan H. N. O’Neill A. K. Soriano P. Bush J. O. (2013). The Widely Used Wnt1-Cre Transgene Causes Developmental Phenotypes by Ectopic Activation of Wnt Signaling. Dev. Biol. 379 (2), 229–234. 10.1016/j.ydbio.2013.04.026 23648512 PMC3804302

[B32] Li B. I. Matteson P. G. Ababon M. F. Nato A. Q. Jr. Lin Y. Nanda V. (2015). The Orphan GPCR, Gpr161, Regulates the Retinoic Acid and Canonical Wnt Pathways during Neurulation. Dev. Biol. 402 (1), 17–31. 10.1016/j.ydbio.2015.02.007 25753732

[B33] Li J. Cui Y. Xu J. Wang Q. Yang X. Li Y. (2017). Suppressor of Fused Restraint of Hedgehog Activity Level Is Critical for Osteogenic Proliferation and Differentiation during Calvarial Bone Development. J. Biol. Chem. 292 (38), 15814–15825. 10.1074/jbc.M117.777532 28794157 PMC5612112

[B34] Lu X. Wang Z. Wang J. Shangguan S. Bao Y. Lu P. (2014). An Association Study betweenSUFUgene Polymorphisms and Neural Tube Defects. Int. J. Neurosci. 124 (6), 436–442. 10.3109/00207454.2013.849249 24070372

[B35] Martínez C. Cornejo V. H. Lois P. Ellis T. Solis N. P. Wainwright B. J. (2013). Proliferation of Murine Midbrain Neural Stem Cells Depends upon an Endogenous Sonic Hedgehog (Shh) Source. PLoS One 8 (6), e65818. 10.1371/journal.pone.0065818 23776550 PMC3679138

[B36] Matteson P. G. Desai J. Korstanje R. Lazar G. Borsuk T. E. Rollins J. (2008). The Orphan G Protein-Coupled Receptor, Gpr161, Encodes the Vacuolated Lens Locus and Controls Neurulation and Lens Development. Proc. Natl. Acad. Sci. 105 (6), 2088–2093. 10.1073/pnas.0705657105 18250320 PMC2538885

[B37] Miyake A. Takahashi Y. Miwa H. Shimada A. Konishi M. Itoh N. (2009). Neucrin Is a Novel Neural-specific Secreted Antagonist to Canonical Wnt Signaling. Biochem. Biophysical Res. Commun. 390 (3), 1051–1055. 10.1016/j.bbrc.2009.10.113 19857465

[B38] Mukhopadhyay S. Wen X. Ratti N. Loktev A. Rangell L. Scales S. J. (2013). The Ciliary G-Protein-Coupled Receptor Gpr161 Negatively Regulates the Sonic Hedgehog Pathway via cAMP Signaling. Cell 152 (1-2), 210–223. 10.1016/j.cell.2012.12.026 23332756

[B39] Nagai‐Tanima M. Hong S. Hu P. Carrington B. Sood R. Roessler E. (2020). Rare Hypomorphic Human Variation in the Heptahelical Domain of SMO Contributes to Holoprosencephaly Phenotypes. Hum. Mutat. 41 (12), 2105–2118. 10.1002/humu.24103 32906187

[B40] Naidich T. P. Altman N. R. Braffman B. H. McLone D. G. Zimmerman R. A. (1992). Cephaloceles and Related Malformations. AJNR Am. J. Neuroradiol. 13 (2), 655–690. 1566723 PMC8333224

[B41] Nasrallah I. Golden J. A. (2001). Brain, Eye, and Face Defects as a Result of Ectopic Localization of Sonic Hedgehog Protein in the Developing Rostral Neural Tube. Teratology 64 (2), 107–113. 10.1002/tera.1052 11460262

[B42] Panhuysen M. Vogt Weisenhorn D. M. Blanquet V. Brodski C. Heinzmann U. Beisker W. (2004). Effects of Wnt1 Signaling on Proliferation in the Developing Mid-/Hindbrain Region. Mol. Cell Neurosci. 26 (1), 101–111. 10.1016/j.mcn.2004.01.011 15121182

[B43] Renard E. Chéry C. Oussalah A. Josse T. Perrin P. Tramoy D. (2019). Exome Sequencing of Cases with Neural Tube Defects Identifies Candidate Genes Involved in One-Carbon/vitamin B12 Metabolisms and Sonic Hedgehog Pathway. Hum. Genet. 138 (7), 703–713. 10.1007/s00439-019-02015-7 31139930

[B44] Rolo A. Galea G. L. Savery D. Greene N. D. E. Copp A. J. (2019). Novel Mouse Model of Encephalocele: post-neurulation Origin and Relationship to Open Neural Tube Defects. Dis. Model. Mech. 12 (11), dmm040683. 10.1242/dmm.040683 31628096 PMC6899037

[B45] Santagati F. Rijli F. M. (2003). Cranial Neural Crest and the Building of the Vertebrate Head. Nat. Rev. Neurosci. 4 (10), 806–818. 10.1038/nrn1221 14523380

[B46] Shanbhag A. G. (1994). Utilization of Information Measure as a Means of Image Thresholding. CVGIP: Graphical Models Image Process. 56(5)**,** 414–419. 10.1006/cgip.1994.1037

[B47] Shimada I. S. Hwang S.-H. Somatilaka B. N. Wang X. Skowron P. Kim J. (2018). Basal Suppression of the Sonic Hedgehog Pathway by the G-Protein-Coupled Receptor Gpr161 Restricts Medulloblastoma Pathogenesis. Cel Rep. 22 (5), 1169–1184. 10.1016/j.celrep.2018.01.018 PMC5813698 29386106

[B48] Shimada I. S. Somatilaka B. N. Hwang S.-H. Anderson A. G. Shelton J. M. Rajaram V. (2019). Derepression of Sonic Hedgehog Signaling upon Gpr161 Deletion Unravels Forebrain and Ventricular Abnormalities. Dev. Biol. 450 (1), 47–62. 10.1016/j.ydbio.2019.03.011 30914320 PMC6497453

[B49] Tabler J. M. Rice C. P. Liu K. J. Wallingford J. B. (2016). A Novel Ciliopathic Skull Defect Arising from Excess Neural Crest. Dev. Biol. 417 (1), 4–10. 10.1016/j.ydbio.2016.07.001 27395007 PMC5023788

[B50] Tang M. Villaescusa J. C. Luo S. X. Guitarte C. Lei S. Miyamoto Y. (2010). Interactions of Wnt/-Catenin Signaling and Sonic Hedgehog Regulate the Neurogenesis of Ventral Midbrain Dopamine Neurons. J. Neurosci. 30 (27), 9280–9291. 10.1523/JNEUROSCI.0860-10.2010 20610763 PMC3578394

[B51] Wang Y. Sun Y. Huang Y. Pan Y. Shi B. Ma J. (2017). The Association Study of Nonsyndromic Cleft Lip with or without Cleft Palate Identified Risk Variants of the $$\varvec{GLI3}$$ G L I 3 Gene in a Chinese Population. J. Genet. 96 (4), 687–693. 10.1007/s12041-017-0808-5 28947718

[B52] Weinert M. Selvakumar T. Tierney T. S. Alavian K. N. (2015). Isolation, Culture and Long-Term Maintenance of Primary Mesencephalic Dopaminergic Neurons from Embryonic Rodent Brains. J. Vis. Exp. (96), 52475. 10.3791/52475 PMC4354654 25741798

[B53] Wilson D. B. Wyatt D. P. (1986). Pathogenesis of Neural Dysraphism in the Mouse Mutant Vacuolated Lens (Vl). J. Neuropathol. Exp. Neurol. 45 (1), 43–55. 10.1097/00005072-198601000-00004 3941326

[B54] Wilson D. B. Wyatt D. P. (1993). *In Vitro* expression of Neural Tube Pathology in the Vl Mutant Mouse. J. Neuropathol. Exp. Neurol. 52 (3), 253–259. 10.1097/00005072-199305000-00009 8492142

[B55] Wu J. Lu X. Wang Z. Shangguan S. Chang S. Li R. (2013). Association between PKA Gene Polymorphism and NTDs in High Risk Chinese Population in Shanxi. Int. J. Clin. Exp. Pathol. 6 (12), 2968–2974. 24294386 PMC3843280

[B56] Xavier G. M. Seppala M. Barrell W. Birjandi A. A. Geoghegan F. Cobourne M. T. (2016). Hedgehog Receptor Function during Craniofacial Development. Dev. Biol. 415 (2), 198–215. 10.1016/j.ydbio.2016.02.009 26875496

[B57] Zhang Z. Wlodarczyk B. J. Niederreither K. Venugopalan S. Florez S. Finnell R. H. (2011). Fuz Regulates Craniofacial Development through Tissue Specific Responses to Signaling Factors. PLoS One 6 (9), e24608. 10.1371/journal.pone.0024608 21935430 PMC3173472

