# Supplementary material for: Wnt1 Lineage Specific Deletion of Gpr161 Results in Embryonic Midbrain Malformation and Failure of Craniofacial Skeletal Development
Source: Front Genet. 2021 Nov 23;12:761418. doi: 10.3389/fgene.2021.761418 (PMC8650154; doi:10.3389/fgene.2021.761418)
Supplement: Supplementary file 1 [file DataSheet1.PDF]

Supplementary Figures and Tables

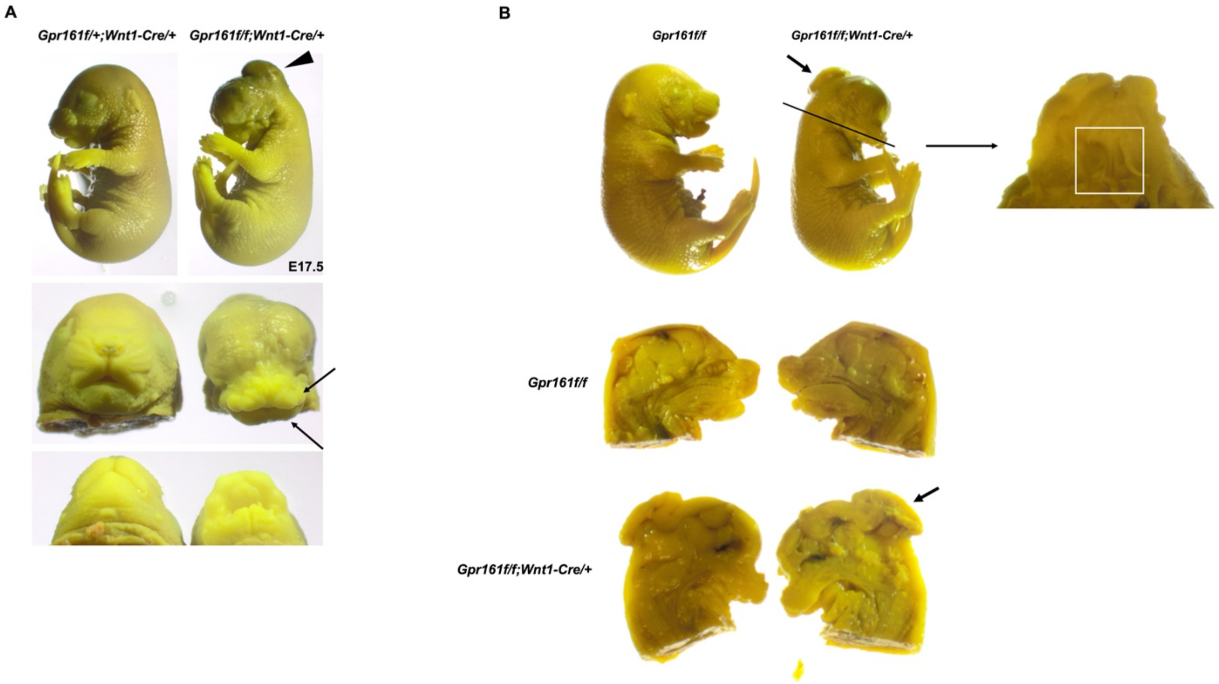

Supplementary Figure 1. The gross morphology **(A)** and sagittal section **(B)** of *Cre* control (*Gpr161*$^{f/+}$*;Wnt1-Cre/*+) and *Gpr161* cKO (*Gpr161*$^{f/f}$*;Wnt1-Cre/*+). The dissected fetuses at E17.5 were fixed in formalin and then Bouin's solution for 24 hrs. (A) Arrowhead indicates the encephaloceles. Arrows indicate widened maxilla and mandible in *Gpr161* cKO. (B) The white box indicates cleft palate in *Gpr161* cKO. Arrows indicate encephaloceles.

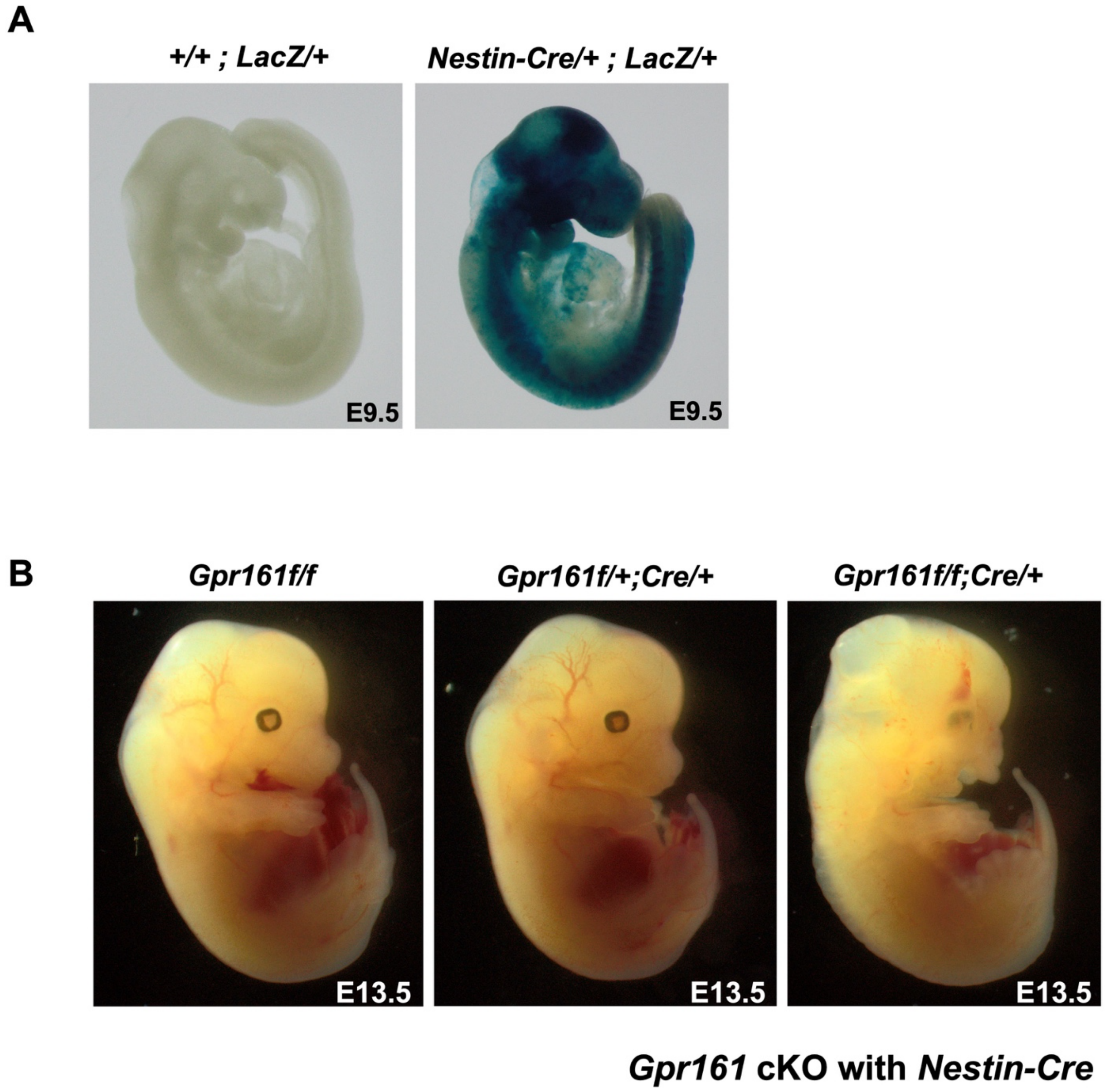

Supplementary Figure 2. Neural stem cell lineage specific deletion of *Gpr161* shows protrusive tectal defects and polydactyly at E13.5. (A) X-gal staining of *Rosa-LacZ;Nestin-Cre/+* and *Rosa-LacZ* at E9.5. **(B)** Gross morphology of *Gpr161* $^{f/f}$, *Gpr161* $^{f/+}$*;Nestin-Cre/+*, *Gpr161*$^{f/f}$*;Nestin-Cre/+* at E13.5.

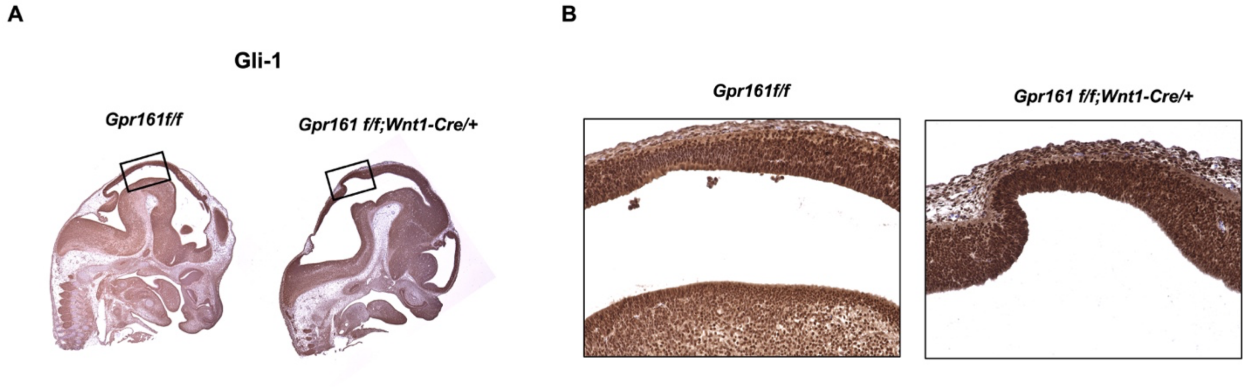

Supplementary Figure 3. IHC with Gli1 in the dorsal midbrain of *Gpr161* cKO fetuses. IHC with Gli1 antibodies in midbrain sections of *Gpr161*$^{f/f}$ and *Gpr161*$^{f/f}$*;Wnt1-Cre/*+ at E13.5 (n=3). The black boxes in (A) indicate the areas magnified in (B).

Supplementary Table 1. The number of malformed fetuses in *Gpr161$^{f/f}$;Wnt1-Cre/+* at E13.5-E18.5

Gpr161$^{f/f}$ x *Gpr161$^{f/+}$;Wnt1-Cre/+* (E13.5-E18.5: 22 litters)					
Genotypes	No of total fetuses	No of fetuses with malformation			
		Tectal hypertrophy	ano/microphthalmia	ano/microtia	orofacial defects
Gpr161$^{f/+}$	40	0	0	0	0
Gpr161$^{f/+}$;Cre/+	54	0	0	0	0
Gpr161$^{f/f}$	51	0	0	0	1 (~1.9%)
Gpr161$^{f/f}$;Cre/+	61	60 (~98%)	60 (~98%)	60 (~98%)	60 (~98%)

Supplementary Table 2. The prevalence of encephalocele in *Gpr161$^{f/f}$;Wnt1-Cre/+* fetuses at E17.5-E18.5

Genotype	No of total fetuses	No of fetuses with malformation	No of fetuses with encephalocele
Gpr161$^{f/f}$;Cre/+	27	26	18 (~69%)

Supplementary Table 3. The number of malformed fetuses in *Gpr161$^{f/f}$;Nestin-Cre/+* at E13.5-E17.5

Gpr161$^{f/f}$ x *Gpr161$^{f/+}$;Nestin-Cre/+* (E13.5-E17.5:10 litters)		
Genotypes	No. of total fetuses	No. of fetuses with malformation (tectal hypertrophy)
Gpr161$^{f/+}$	16	0
Gpr161$^{f/+}$;Cre/+	24	0
Gpr161$^{f/f}$	20	0
Gpr161$^{f/f}$;Cre/+	19	19 (100%)
